# A study of latent profile analysis of empathic competence and factors influencing it in nursing interns: a multicenter cross-sectional study

**DOI:** 10.3389/fpubh.2024.1434089

**Published:** 2024-06-26

**Authors:** Jia Wang, Xiaoqing Xu, Jiaxin Sun, Yujia Ma, Peijuan Tang, Wenzhong Chang, Xia Chen, Yajuan Cui, Mei Su, Yan He

**Affiliations:** ^1^Department of Gynaecology, Inner Mongolia People's Hospital, Hohhot, China; ^2^School of Nursing, Inner Mongolia Medical College, Hohhot, China; ^3^Department of Clinical Medical Research Center, Affiliated Hospital of Inner Mongolia Medical University, Hohhot, China; ^4^STD/AIDS Prevention and Control Section, Tongliao Center for Disease Control and Prevention, Tongliao, China; ^5^Department of Nursing, Baotou Central Hospital, Hohhot, China

**Keywords:** empathy, nursing interns, latent profile analysis, professional quality of life scale, China

## Abstract

**Background:**

Empathy, as one of the fundamental principles of nursing professionalism, plays a pivotal role in the formation and advancement of the nursing team. Nursing interns, as a reserve force within the nursing team, are of significant importance in terms of their ability to empathize. This quality is not only directly related to the degree of harmony in the nurse–patient relationship and the enhancement of patient satisfaction, but also plays a pivotal role in the promotion of the quality of nursing services to a new level.

**Aim:**

The objective of this study was to gain a deeper understanding of the current state of nursing interns’ empathic abilities. To this end, we sought to examine empathic performance under different profile models and to identify the key factors influencing these profile models.

**Methods:**

The study utilized 444 nursing interns from 11 tertiary general hospitals in Inner Mongolia as research subjects. The study employed a number of research tools, including demographic characteristics, the Jefferson Scale of Empathy, and the Professional Quality of Life Scale. A latent profile model of nursing interns’ empathy ability was analyzed using Mplus 8.3. The test of variability of intergroup variables was performed using the chi-square test. Finally, the influencing factors of each profile model were analyzed by unordered multi-categorical logistic regression analysis.

**Results:**

The overall level of empathy among nursing interns was found to be low, with 45% belonging to the humanistic care group, 43% exhibiting low empathy, and 12% demonstrating high empathy. The internship duration, empathy satisfaction, secondary traumatic stress, only child, place of birth, and satisfaction with nursing were identified as factors influencing the latent profiles of empathy in nursing interns (*p* < 0.05).

**Conclusion:**

There is considerable heterogeneity in nursing interns’ ability to empathize. Consequently, nursing educators and administrators should direct greater attention to interns with lower empathy and develop targeted intervention strategies based on the influences of the different underlying profiles.

## 1 Introduction

Empathy is widely recognized as a necessity for effective nursing care and a core element of quality care (1). According to the ICN (2), empathy is the foundation of the nursing profession and the key to achieving more humane, effective, and inclusive healthcare. A lack of effective empathy skills can result in a tense and challenging relationship between nurses and patients in clinical settings, which can make nurses the primary target of nursing complaints (3). The enhancement of nurses’ empathy abilities can facilitate nurse–patient communication and exchange, enhance patients’ trust in nurses, reduce nurses’ burnout, and contribute to the improvement of nursing care quality (4).

As the core force of the future nursing workforce, the level of empathic competence of nursing students directly predicts the quality of future nursing care and the overall level of development. To address this, many studies have been conducted. The worrying findings of Korkmaz Doğdu et al. (5) revealed a general low level of empathy among nursing students. This finding not only affects the effective communication between students and patients in clinical practice, but also inadvertently exacerbates the gap and misunderstanding between nurses and patients. Meanwhile, Xu et al.’s (6) study further revealed the chain reaction caused by the decline in empathy, in which the significant increase in emotional stress was particularly prominent. In the face of the complex emotions and challenges of daily nursing work, the lack of empathy made it difficult for students to cope, which not only affected their physical and mental health in the long run, but may also lead to burnout and a gradual loss of enthusiasm and motivation for nursing work (7). It is worth noting, however, that nursing students’ empathy skills are not static. Their empathic skills continued to improve during the course of their studies from matriculation to the third year, but generally declined after entering the clinical placement phase (8). This dramatic change in empathic ability undoubtedly has a serious impact on the state of nursing students’ relationships with patients, which in turn poses a threat to the quality of nursing care. Therefore, we need to explore in depth the risk factors that affect nursing interns’ empathic ability and seek effective measures to improve nursing interns’ empathic ability to ensure that they can better serve patients in their future nursing work.

Several studies have given us clues. A study (9) found that certain factors within the interns themselves, such as gender, age, and marital status, may have some impact on their empathic abilities. Specifically, male interns demonstrated higher levels of empathy in the area of perspective taking, and they were better at examining situations from different angles to gain a deeper understanding of others’ perspectives. Comparatively, female interns scored higher on empathic concern, meaning that they were more likely to have deep empathy and concern for others. The results of this study reveal the differentiated performance of different genders in terms of empathic ability, providing deeper insights. In addition, another study (10) found that nursing interns are exposed to a large number of patients during their clinical practicum, and this increase in patient care may lead to a decrease in their empathic abilities. Meanwhile, a further study (11) indicated that complex interpersonal relationships would also have a significant impact on empathy. When dealing with complex interpersonal relationships, interns need to be more sensitive to sensing and understanding the changing emotions of others. This ability to sense and understand emotions is an important part of empathy. The above findings suggest that the decline in empathy not only affects nursing interns’ effective communication with patients, but may also lead to their inability to truly feel the pain and distress of patients, thus preventing them from providing effective psychological support and comfort, and may even lead to medical disputes and complaints (12).

Currently, there are a variety of approaches to studying empathy in nursing interns: Von Knorring et al. (13) used a qualitative study to explore nursing interns’ empathy, which was a good way to describe interns’ empathic experiences on a subjective level, but this approach lacked the support of objective data; Chen et al. (10) study, in contrast to the previous one, used objective statistics, which clearly illustrated the high and low levels of nursing interns’ empathy and their related factors, but this approach evaluated empathy only on the basis of scale scores, ignoring the heterogeneity of different profiles of empathy. In contrast to the above research methods, Latent Profile Analysis (LPA) is used to determine the latent characteristics of an individual based on his or her response patterns on the exoteric topics, to understand the percentage of the number of people in each latent profile, and to further identify the heterogeneity that exists in the sample (14). This technique is particularly useful for analyzing diagnoses with a high degree of heterogeneity, such as borderline personality disorder (15) and empathy (16), where LPA can effectively discriminate between individuals in terms of differences between outlier items on the scale, allowing the construction of a model for targeted intervention. There have been numerous applications in the field of nursing (17–20), which well illustrate the current status of different profiles of nurse empathy and what are the influencing factors. However, few researchers have applied it to nursing interns as a group. Compared with nurses, nursing interns are also an indispensable backbone in clinical work, and their empathic ability will greatly affect the quality of nursing work.

Therefore, this study explores the potential profiles of nursing interns’ empathy through potential profile analysis and analyzes the population characteristics and influencing factors of different profiles to provide a new reference base for nursing educators and administrators to develop a comprehensive and targeted empathy development program.

## 2 Methods

### 2.1 Subjectivity and data collection

#### 2.1.1 Subjectivity

This study is a multicenter cross-sectional study. A convenient sampling method was used to select 444 nursing interns who were practicing in 11 tertiary general hospitals in Inner Mongolia from September 2023 to February 2024.The inclusion criteria were as follows: (1) full-time nursing students in general medical colleges and universities across China; (2) undergoing professional internship in the clinical department of the hospital, and the internship duration was≥3 months; (3) informed consent and voluntary participation in the study. Those who did not have consecutive internships in the same hospital were excluded from the study. According to Kendall’s criterion, the sample size in a regression sample is at least 10 times the number of independent variables, and the number of independent variables in this study was 22, and considering 20% of invalid responses, the minimum sample size was N = 22 × 10 + [(22 × 10) × 20%] = 264. The final effective sample size included in this study was 444 cases. See Supplementary Material for a sampling description.

#### 2.1.2 Data collection

We collected data using a mobile phone questionnaire star mini program. After the questionnaire was created, the mini-program generated a two-dimensional code, and the investigators asked participants to carefully review the informed consent form and then complete the questionnaire anonymously.

### 2.2 Measures

The questionnaires used in this study included: socio-demographic characteristics, Jefferson Scale of Empathy and Professional Quality of Life Scale (ProQOL). All questionnaires were reviewed by five professors in the field and then used.

#### 2.2.1 Socio-demographic characteristics

Self-designed after a preliminary review of the literature. These include age, gender, only child, family situation, place of birth, monthly disposable living expenses, internship duration, educational level, school grades, student leader, part-time work experience, whether the nursing program is the first choice, and satisfaction with nursing.

#### 2.2.2 Jefferson scale of empathy

The Jefferson Scale of Empathy was developed by Mohammadreza Hojat and his research team at Jefferson University, USA, to measure empathy in healthcare workers. The scale consists of 20 items and 3 dimensions (viewpoint selection, emotional care, and transpersonal thinking), of which the 2 dimensions of emotional care and transpersonal thinking are reverse scored. The scale was scored on a 7-point Likert scale, with positive items ranging from “strongly disagree” to “strongly agree” and scored from 1 to 7, while negative items were scored in reverse, with higher total scores indicating greater empathy. This study used the Chinese version of the scale, which had a Cronbach’s alpha coefficient of 0.836, reflecting good reliability and validity (21). In this study, the overall Cronbach’s alpha coefficient of the questionnaire was 0.832, the folding reliability was 0.724, the viewpoint selection dimension was 0.870, the emotional care dimension was 0.873, and the transpersonal thinking dimension was 0.735, indicating that the scale had good internal consistency.

#### 2.2.3 Professional quality of life scale, ProQOL

The scale was revised by Stamm (22), and the Chinese version of the Professional Quality of Life Scale was used in this study. The scale consists of three dimensions: empathy satisfaction, burnout, and secondary traumatic stress, with 10 items for each dimension and a total of 30 items. And it was scored on a 5-point Likert scale from “never” to “always.” In this study, the overall Cronbach’s alpha coefficient of the questionnaire was 0.941, the half reliability was 0.867, the empathy satisfaction dimension was 0.941, the burnout dimension was 0.786, and the secondary traumatic stress dimension was 0.903, indicating that the scale had good internal consistency.

### 2.3 Statistics methods

A latent profile model of nursing interns’ empathy was analyzed using Mplus 8.3. The 20-item score of the Jefferson Scale of Empathy was used as an exogenous variable, and profiles from 1 to 5 were sequentially selected for fitting. Akaike Information Criterion (AIC), Bayesian Information Criterion (BIC), and adjusted Bayesian Information Criterion (aBIC) are among the information metrics, and smaller values of the three metrics indicate better model fit; Lo–Mendell–Rubin (LMR) and Bootstrapped Likelihood Ratio Test (BLRT) are used to compare k-1 and k differences in fit between model profiles; Entropy is between 0 and 1, with larger values indicating better model fit (≥0.8 corresponds to 90% of cases correctly classified). Data were checked using Excel 2019 and analyzed using SPSS 24.0. Enumeration data are expressed as frequencies and percentages, and measurement data are described as mean ± standard deviation. Tests of variability of variables between groups were performed using the chi-squared test. Unordered multi-categorical logistic regression analysis was performed with the adjusted latent profiles as the dependent variable and general information and scores on each dimension of the Professional Quality of Life Scale as the independent variables. The test level was α = 0.05 (two-tailed).

### 2.4 Ethical statement

The study was approved by the Ethics Committee of the Inner Mongolia People’s Hospital (Ethics No. 202405805 L). All participating nursing interns gave informed consent before completing the electronic version of the questionnaire. The respondents’ personal information was kept strictly confidential, and their right to privacy was protected in accordance with the Declaration of Helsinki (23).

## 3 Results

### 3.1 Participant characteristics

A total of 444 nursing interns were studied in this study, 362 females and 82 males, the average age was 21.82 ± 2.56 years and the average internship duration was 4.30 ± 1.57 months. 165 were only children, 112 were student leaders and 307 had their first choice of nursing program. Family situation: 393 were two parents, 39 were single parents and 12 were remarried. Place of birth: 259 were in rural areas, 121 were in towns, and 64 were in urban areas. Monthly disposable living expenses: 373 ≤ 2000 yuan. Educational level: 188 undergraduates, 256 specialties. School grades: 31 in the top three of the grade, 395 in the middle, and 18 in the poor. 155 had no experience of part-time work, 210 had occasional part-time work, and 79 had part-time work many times. 78 were very satisfied with the nursing work, 179 were satisfied, 162 were generally satisfied, 13 were dissatisfied, and 12 were very dissatisfied.

### 3.2 Nursing interns’ empathy scores

**Figure 1:**
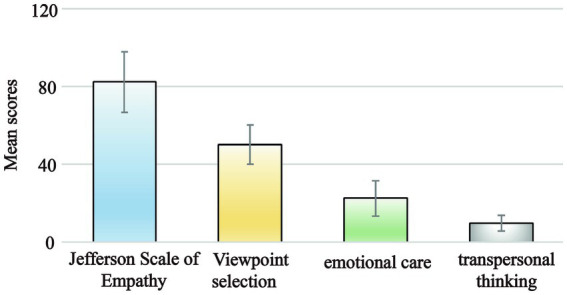
Nursing interns’ Jefferson scale of empathy scores.

Nursing interns’ empathy score was 82.18 ± 15.55, with viewpoint selection 50.07 ± 10.10, emotional care 22.51 ± 8.96 and transpersonal thinking 9.59 ± 4.02, as shown in Figure 1.

### 3.3 Results of empathy latent profile fitting for nursing interns

**Figure 2:**
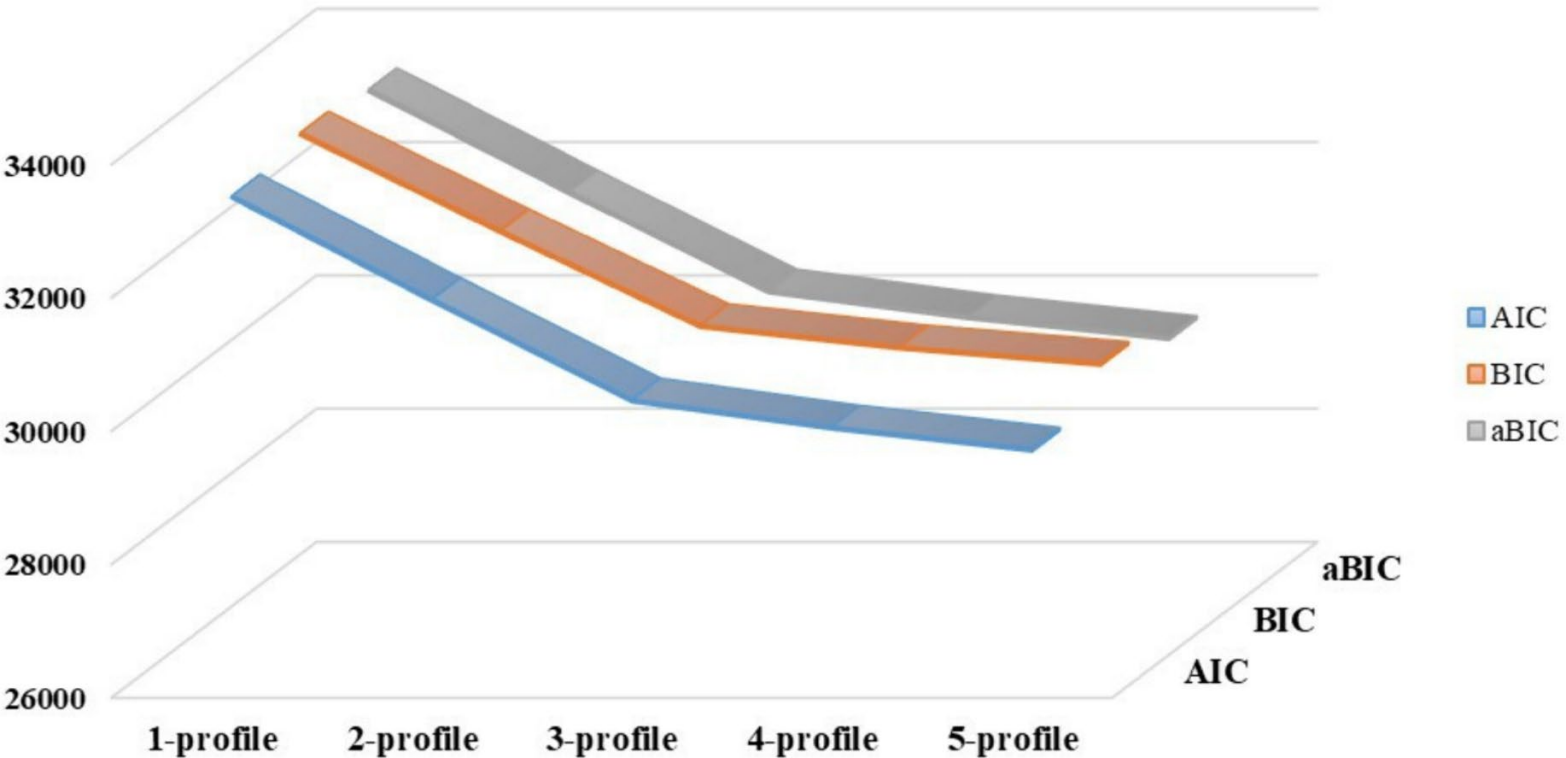
Five model parameter variations.

Using the 20 items of the Jefferson Scale of Empathy as exogenous indicators, 1 to 5 latent profiles were sequentially selected for exploratory latent profile analysis of nursing interns’ empathy (Figure 2), and the results showed that AIC, BIC, and aBIC kept decreasing with the increase of the profiles, and the mean value of Entropy was above 0.8, and the most optimal value of Entropy was obtained when 3 profiles were retained, and LMR and BLRT reached a significant level. Therefore, 3 profiles were selected on a comprehensive basis, as shown in Table 1.

**Table 1:** Fit indices for latent profile analysis (*n* = 444).

Latent Profile	AIC	BIC	aBIC	LMR (p)	BLRT (p)	Entropy	Group size for each profile	Proportion of each profile
1-profile	33287.29	33451.12	33324.18				444	
2-profile	31734.97	31984.81	31791.22	0.0047	<0.001	0.906	209/235	0.471/0.529
3-profile	30225.03	30560.89	30300.65	<0.001	<0.001	0.947	198/191/55	0.446/0.430/0.124
4-profile	29816.18	30238.05	29911.18	0.0099	<0.001	0.921	143/178/67/56	0.322/0.401/0.151/0.126
5-profile	29485.17	29993.05	29599.53	0.3798	<0.001	0.926	54/116/164/61/49	0.122/0.261/0.369/0.137/0.110

AIC, Akaike Information Criterion; BIC, Bayesian Information Criterion; aBIC = adjusted BIC; LMR, Lo–Mendell–Rubin adjusted likelihood ratio test for K vs. K-1 profiles; BLRT, Bootstrapped Likelihood Ratio Test.

### 3.4 Classifying and naming of empathy in nursing interns

**Figure 3:**
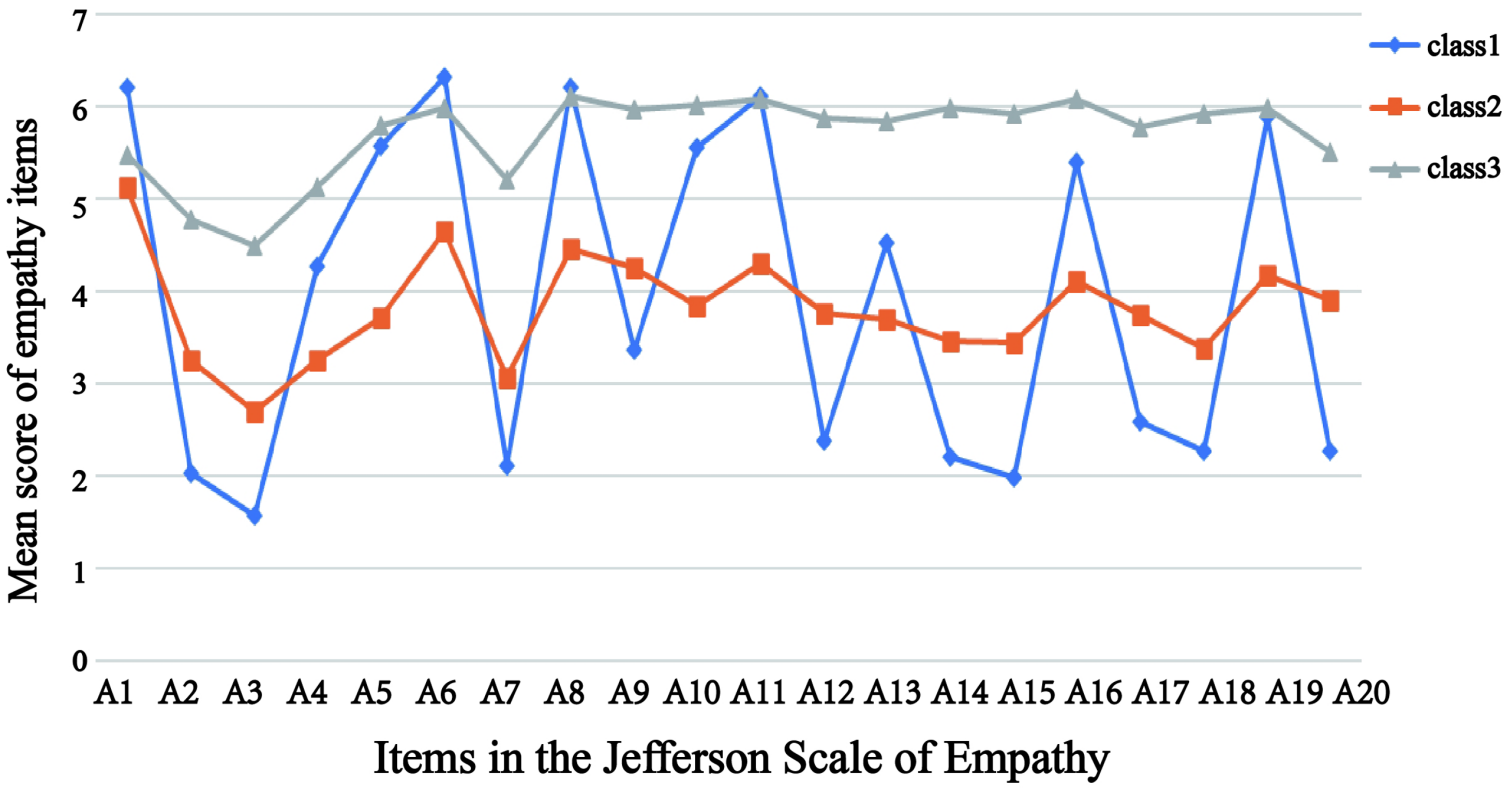
Response to each items in the 3 potential profiles of nursing interns empathy.

Based on the three profile models obtained from the latent profile analysis, the responses of C1, C2, and C3 on the empathy scale were plotted (Figure 3). The horizontal coordinates represent the 20 items in the scale, and the vertical coordinates represent their corresponding means. The higher the score of each item, the better the individual’s ability to empathize, and also combined with the fluctuation of the item scores, the profiles were named: C1 was at a high level in the original scale item 1 (It is important to understand the emotional state of the patient and family), item 6 (It is as important to understand the patient’s body language as it is to communicate verbally), item 8 (Paying attention to the patient’s body language and nonverbal cues), item 11 (Considering the patient’s point of view), and item 19 (Thinking from the patient’s standpoint), interns in this group were able to carefully observe and interpret patients’ nonverbal messages, fully consider patients’ perspectives, and were adept at thinking from the patient’s perspective. Thus this profile was defined as the humanistic care group, a total of 198 (45%); C2 was at a lower level in the original scale items 1 (It is important to understand the emotional state of the patient and family), item 4 (Lack of empathy makes it difficult to be a successful nurse), item 5 (Feeling empathy for the patient makes them feel better), item 8 (Paying attention to the patient’s body language and nonverbal cues), item 10 (Empathy is an important factor in the treatment process), item 11 (Thinking about the patient’s perspective), 13 (Feeling empathy for the patient makes them feel that treatment is effective), item 16 (Humor helps patients get better clinical outcomes), and item 19 (Feeling humor helps patients get better clinical outcomes), this group showed less sensitivity and concern for patients’ emotions, needs, and feelings in medical care. Therefore this profile was defined as the low empathy group, with a total of 191 (43%); C3’s total score was higher than the other two profiles, this group of interns are able to deeply understand and pay attention to the emotional state of patients and their families, are good at picking up on patients’ body language and nonverbal cues, and demonstrate a high level of sensitivity and humanistic care in medical care. So it was defined as the high empathy group, with a total of 55 (12%).

### 3.5 Distributional characteristics of latent profiles of empathy in nursing interns

The results of the univariate analysis showed that the differences in the distribution of the latent profiles of the nursing interns of place of birth, educational level, whether the nursing program is the first choice and satisfaction with nursing were statistically significant (*p* < 0.05), as shown in Table 2.

**Table 2:** Comparison of the distribution of potential profiles of empathy among nursing interns with different socio-demographic characteristics.

Characteristic	Class1 (*n* = 198)	Class2 (*n* = 191)	Class3 (*n* = 55)	chi-square	*p*
Place of birth					
Rural areas	126 (63.6)	103 (53.9)	30 (54.5)	10.598	0.031
Town	51 (25.8)	59 (30.9)	11 (20.0)		
Urban areas	21 (10.6)	29 (15.2)	14 (25.5)		
Educational level					
Undergraduate	101 (51.0)	59 (30.9)	28 (50.9)	18.007	<0.001
Specialty	97 (49.0)	132 (69.1)	27 (49.1)		
Whether the nursing program is the first choice					
Yes	151 (76.3)	119 (62.3)	37 (67.3)	8.982	0.011
No	47 (23.7)	72 (37.7)	18 (32.7)		
Satisfaction with nursing					
Very satisfied	40 (20.2)	25 (13.1)	13 (23.6)	38.102	<0.001
Satisfied	98 (49.5)	59 (30.9)	22 (40.0)		
Generally satisfied	57 (28.8)	86 (45.0)	19 (34.5)		
Dissatisfied	3 (1.5)	10 (5.2)	0 (0)		
Very dissatisfied	0 (0)	11 (5.8)	1 (1.8)		

Unit: persons (%). Only statistically significant variables are listed.

### 3.6 A multifactorial analysis of factors influencing latent profiles of empathy in nursing interns

The factors that were statistically significant in the univariate analyses as well as the dimensions in the Professional Quality of Life Scale were used as independent variables, and the three latent profiles of empathy were used as dependent variables in an unordered multi-categorical logistic regression analysis (Table 3). Using the high empathy group as a reference, internship duration, empathy satisfaction, secondary traumatic stress, only child, place of birth and satisfaction with care are the influencing factors for the humanistic care group; empathy satisfaction, secondary traumatic stress and burnout are the influencing factors for the low empathy group.

**Table 3:** Multifactorial analysis of factors influencing potential profiles of empathy in nursing interns (Logistic regression).

Profiles	Variables	B	SE	Wald chi-square	P	OR	95%CI
C1 vs. C3 (C3 as a reference)	Constant	25.843	3614.706	0.000	0.994		
	Internship duration	−0.285	0.122	5.424	0.020	0.752	[0.592, 0.956]
	empathy satisfaction	0.250	0.052	23.112	0.000	1.284	[1.160, 1.422]
	Secondary traumatic stress	−0.222	0.055	16.308	0.000	0.801	[0.719, 0.892]
	Only child	−0.918	0.455	4.067	0.044	0.399	[0.164, 0.975]
	Place of birth(urban areas)	1.942	0.604	10.326	0.001	6.973	[2.133, 22.794]
	Satisfaction with nursing(dissatisfied)	−15.609	0.848	339.147	0.000	1.664E-07	[3.161E-08, 8.763E-07]
C2 vs.C3 (C3 as a reference)	Constant	46.584	3524.738	0.000	0.989		
	Empathy satisfaction	0.119	0.051	5.510	0.019	1.126	[1.020, 1.244]
	Burnout	−0.171	0.078	4.860	0.027	0.843	[0.724, 0.981]
	Secondary traumatic stress	−0.119	0.055	4.754	0.029	0.888	[0.798, 0.988]

OR, odds ratio; 95% CI, confidence interval.

Independent variable assignment, place of birth, rural areas = (0,0,0), town = (0,1,0), urban areas = (0,0,1); educational level, undergraduate = 1, specialty = 2; whether the nursing program is the first choice, yes = 1, no = 2; satisfaction with nursing, very satisfied = (0,0,0,0,0,0), satisfied = (0,1,0,0,0), generally satisfied = (0,0,1,0, 0), dissatisfied = (0,0,0,1,0), and very dissatisfied = (0,0,0,0,0,1). The results of the analysis showed that internship duration, empathy satisfaction, secondary traumatic stress, only child, place of birth, satisfaction with nursing and burnout are the influencing factors of empathy in nursing interns.

Although this study provided insights into the empathy of nursing interns through profile analysis and identified the factors influencing each profile, there are still some limitations that need to be pointed out here. First, the sample of this study may not have been broad enough to include only interns in a particular region, which may limit the generalizability of the findings. Future research could expand the sample to provide a more comprehensive understanding of the empathy skills of nursing interns from different backgrounds. Second, the instrument used to measure empathy skills in this study may have limitations; although every effort was made to select widely accepted assessment tools, different measurement tools may yield different results. Future research could explore multiple measurement tools to validate the findings of this study. In addition, this study may not have accounted for all possible variables when examining influences on empathy. For example, factors such as interns’ personality traits and family background may have an impact on empathy skills, but this study did not examine each of these factors individually. Future research could further explore the relationship between these variables and empathy skills. Finally, due to the cross-sectional design of this study, it was not possible to determine the trend of empathy ability over time. Future studies could use a longitudinal design to track the dynamics of empathic competence in nursing students to gain a deeper understanding of the developmental process of empathic competence.

## 4 Discussion

### 4.1 Nursing interns are heterogeneous in their ability to empathize

#### 4.1.1 Interpretation of results

This study enriched previous findings on empathy among nursing interns by identifying three different profiles among nursing interns, namely humanistic care group (78.91 ± 7.36), low empathy group (76.36 ± 10.74) and high empathy group (114.13 ± 13.46). The scores for the humanistic care group indicate that the nursing interns demonstrated a moderate level of empathy in humanistic care. This indicates that the interns were able to understand and care for their patients, but there may be room for improvement. The scores of the low empathy group indicate that these nursing interns lack empathy and may have difficulty fully understanding and feeling the emotions and needs of their patients, which may affect their ability to provide personalized care, revealing that the empathy of some of the nursing interns needs to be improved. The scores of the high empathy group indicate that these interns excel in empathy. They have high levels of empathy and are able to deeply understand the feelings and needs of their patients, which is essential to providing quality, personalized care. These highly empathetic interns can also serve as role models and trainers to help other interns improve their empathy skills. Overall, the scores of these three groups reflect the differences in empathic competence among nursing interns and reveal areas for attention and improvement in nursing education. Through targeted education and training, the empathy of nursing interns can be improved, thereby enhancing the quality of nursing care (9).

#### 4.1.2 Comparison with literature

The results of the study showed that the empathy score of nursing interns was 82.18 ± 15.55, which was lower than the findings of Li et al. (24) and Yu et al. (25). The reason for this analysis may be related to the difference in the study population, which were freshmen and sophomores, respectively, while the population of the present study was nursing students at the internship stage. In addition, different healthcare organizations may have different work atmospheres and requirements, all of which may affect an intern’s psychological state and empathy performance. The researcher may have inadvertently introduced subjective biases, such as expectations or preferences for certain outcomes, during data collection and analysis. The stresses and challenges of the clinical internship may cause them to focus more on the technical aspects of the job and relatively less on the emotional aspects of the patient. It has been shown that medical students’ empathy decreases after the third year of study (26), which further confirms that empathy is generally low among students in the internship phase. A survey found that interns who do not receive adequate attention, guidance and support during their internship may feel lonely and helpless, which is not conducive to the development and maintenance of empathic competence (9).

#### 4.1.3 Practical implications

Therefore, schools and hospitals should pay attention to interns’ work stress by providing adequate psychological support, improving the clinical environment, enhancing teamwork, and strengthening interns’ empathy satisfaction, such as implementing regular counseling, establishing support groups, and optimizing the scheduling system in order to help them better understand and cope with the challenges and conflicts in the clinical environment (27).

#### 4.1.4 Future research

Future research could consistently track changes in nurse interns’ empathic competence across stages of the practicum experience to understand the impact of the practicum experience on the development of empathic competence.

### 4.2 Differences in factors influencing different latent profiles of empathy In nursing interns

#### 4.2.1 The internship duration - impact factors of empathy

##### 4.2.1.1 Interpretation of results

The internship duration is an influential factor in the empathic competence of nursing interns. Our study found that, the longer the internship duration, the humanistic care group tended to have lower levels of empathy.

##### 4.2.1.2 Comparison with literature

As the internship duration progresses, nursing interns may gradually shift from the initial novelty and empathy to a more professional, task-oriented mindset. They may become more focused on completing clinical tasks and meeting job requirements, and pay relatively little attention to the emotional needs of patients. This change in role perception and coping style may decrease their ability to empathize (28).

##### 4.2.1.3 Practical implications

For such students, clinical departments can appropriately adjust the teaching mode of interns and introduce novel teaching methods to alleviate interns’ fatigue and improve their empathy. For example, the application of PBL, CBL teaching method (29), and video feedback combined with peer role-playing (30) to clinical teaching of nursing interns can not only enrich the ways of communicating with others, accepting more diversified ideas and concepts, and broadening their knowledge, but also help interns effectively improve their psychological condition and their empathic state (31). In addition, Söderberg et al. (32) used role-playing to enable nursing students to talk to patients and experience eye contact, so that they can relate to others and make boring knowledge come alive, which can also promote the improvement of empathy.

##### 4.2.1.4 Future research

Future research could further refine the study to examine the specific relationship between the length of the internship and the decline in empathy. For example, is there a tipping point beyond which an intern’s ability to empathize declines significantly? Such a study could help determine the optimal internship cycle to balance the improvement of professional skills with the maintenance of empathy.

#### 4.2.2 Place of birth - impact factors of empathy

##### 4.2.2.1 Interpretation of results

Place of birth is an influential factor in nursing interns’ ability to empathize. This study showed that interns from urban areas were more likely to be in the humanistic care group than interns from rural areas and towns. The urban environment tends to be more diverse and open, so urban nursing interns are more likely to be exposed to people from different backgrounds and cultures (33).

##### 4.2.2.2 Comparison with literature

In contrast, the study by Jia-Ru et al. (34) showed that interns from cities were less able to empathize than interns from rural areas. The reason for this may be analyzed as follows: the methodological differences between the present study and JIA-RU J’s study may be an important reason for the different conclusions. Systematic reviews provide comprehensive, objective evidence to assess the impact of the urban environment on nursing interns’ empathy, whereas cross-sectional studies are more focused on describing phenomena at a specific point in time. In addition, urban environments offer more cultural diversity and interpersonal opportunities that can help develop humanistic nursing skills in practicing nurses. However, as JIA-RU J points out, urban environments can also lead to cultural clashes and divisions that can confuse practicing nurses when dealing with complex cultural interactions, which in turn affects their empathy.

##### 4.2.2.3 Practical implications

For interns from rural areas, educational institutions can actively create more opportunities for them to be exposed to urban multicultural environments, such as arranging internships in urban healthcare organizations, to enhance their cultural adaptation and empathy.

##### 4.2.2.4 Future research

Future research could further explore how factors such as different geographic cultures and socioeconomic status influence the development of empathy in practicing nurses.

#### 4.2.3 Only child - impact factors of empathy

##### 4.2.3.1 Interpretation of results

Only child is an influencer of nursing interns’ empathy. It was found that only child has lower empathy than non-only child.

##### 4.2.3.2 Comparison with literature

It was found that only child has lower empathy than non-only child, which was confirmed in the study by Zhai et al. (35). Only child often grow up in a different environment than non-only child. They may not have siblings in their families with whom to share their life experiences and emotional interactions, which may result in relatively less experience in dealing with interpersonal relationships and emotional exchanges (36). Nursing requires a great deal of emotional involvement and understanding of others during practice, and only child may appear to be relatively deficient in this area.

##### 4.2.3.3 Practical implications

Therefore, educators should strengthen emotional communication with only child, encourage them to express their emotions, and learn to listen to and understand the emotions of others. For example, interns can keep an emotional diary, attend emotional counseling sessions, have regular one-on-one sessions, etc.

##### 4.2.3.4 Future research

Future research could look more closely at how only child’s home environments, including factors such as parenting styles and family atmosphere, affect their ability to empathize. It could also compare the differences in family environments between only child and non-only child and analyze how these differences lead to differences in empathy.

#### 4.2.4 Empathic satisfaction - impact factors of empathy

##### 4.2.4.1 Interpretation of results

Empathic satisfaction is an influential factor of nursing interns’ empathic competence. The logistic regression results showed that the higher the empathic satisfaction of the interns, the higher the empathic competence of the humanistic care group and the low empathy group.

##### 4.2.4.2 Comparison with literature

As the interns’ empathy satisfaction increased, so did the empathy of the humanistic and low empathy groups, which is consistent with the results of Cao et al. (37). When interns are satisfied with their empathy, they may be more inclined to actively establish emotional relationships with patients and understand their needs and feelings, thus further improving their empathy (38).

##### 4.2.4.3 Practical implications

Therefore, before the beginning of the internship and during the internship process, internship organizations can organize special empathy competency training courses to help interns understand the importance of empathy and learn how to effectively listen to, express, and understand patients’ emotions. For example, various training methods such as role-playing, standardized patient interviews, interactions with real patients, and video recording of interviews have been shown to be effective in improving students’ empathy (39). Through these hands-on training methods, students can not only understand and experience the situation and emotions of patients in real life, but also practice their communication skills and emotional empathy in real life, thus better cultivating and developing their empathy.

##### 4.2.4.4 Future research

Future research could further examine specific indicators of empathic satisfaction and how these quantitatively affect nursing interns’ ability to empathize.

#### 4.2.5 Secondary traumatic stress - impact factors of empathy

##### 4.2.5.1 Interpretation of results

Secondary traumatic stress is an influential factor in the empathic competence of nursing interns. Our study revealed that interns with low secondary traumatic stress scores were more likely to be in the humanistic care group and the low empathy group, when nursing interns were less exposed to secondary traumatic stress, they were better able to maintain their emotional stability and thus were more willing to attend to their patients’ emotional needs and provide humanistic care.

##### 4.2.5.2 Comparison with literature

The finding is consistent with the results of a study by Cao et al. (37). At the same time, the low empathy group does not imply a lack of compassion and caring, but may be due to their ability to maintain some emotional distance and avoid over-involvement when dealing with traumatic experiences. Therefore, moderate secondary traumatic stress is important for nursing interns (40). They need to maintain a level of sensitivity when dealing with traumatic patient experiences, but they also need to learn to manage their emotions and avoid overinvolvement or indifference.

##### 4.2.5.3 Practical implications

By developing appropriate empathy and emotional management skills, such as the ability to listen, to think differently, to regulate emotions, nursing interns can better adapt to the practice environment and provide quality care.

##### 4.2.5.4 Future research

To examine trends in secondary traumatic stress at different stages of nursing practice and its dynamic impact on empathic competence. In addition, to analyze in depth the relationship between secondary traumatic stress and empathic competence, including whether there is a threshold effect, i.e., secondary traumatic stress reaches a certain level before it significantly affects empathic competence.

#### 4.2.6 Burnout - impact factors of empathy

##### 4.2.6.1 Interpretation of results

Burnout is an influential factor in the empathic competence of nursing interns. When interns experience burnout, it further exacerbates the lack of empathy in the low empathy group.

##### 4.2.6.2 Comparison with literature

This result is consistent with the findings of Williams et al. (41) and Melnick and Powsner (42). Burnout is usually characterized by emotional exhaustion, loss of interest and enthusiasm for work, and possible interpersonal tension (43). These emotional and psychological stresses can sap interns’ energy and make it difficult for them to devote sufficient emotion and attention to their work. In this case, even residents who already have low levels of empathy may find it more difficult to understand and feel patients’ emotions and needs due to burnout.

##### 4.2.6.3 Practical implications

Therefore, it is necessary to pay special attention to the psychological state of interns in the process of internship, and to recognize and deal with the problem of burnout in time to promote the improvement of their empathic ability and overall development.

##### 4.2.6.4 Future research

Future research could continue to track changes in nursing interns’ burnout and empathic skills over the course of their internship. Periodic assessments could be used to understand how the development of burnout affects interns’ empathic performance and whether this effect changes as the internship progresses.

#### 4.2.7 Nursing job satisfaction - impact factors of empathy

##### 4.2.7.1 Interpretation of results

Nursing job satisfaction is an influencing factor of nursing interns’ empathy. Nursing work requires a high degree of responsibility and patience, and interns are often required to acquire a large amount of knowledge and skills in a short period of time, as well as to deal with a variety of emergencies, and these pressures may lead to interns’ job dissatisfaction and, consequently, low empathic ability.

##### 4.2.7.2 Comparison with literature

The results of the present study are consistent with the findings of Hamaideh et al. (44) and Kim (11). The above reminds us to observe and pay attention to what interns think, know, and do in their education and development efforts, and to screen for interns with low empathy. Cognitively and emotionally, we should help interns understand, share, and engage in the patient’s situation, and help each intern recognize the importance of empathy for themselves and their patients.

##### 4.2.7.3 Practical implications

Importantly, nursing educators in schools and hospitals should focus on debriefing and reflection after experiential/immersive learning, both of which are catalysts for empathy and can unconsciously improve interns’ empathy (45, 46).

##### 4.2.7.4 Future research

Future research could compare the differences in empathy between high and low satisfaction nursing interns and explore the specific impact of satisfaction on empathic ability. For example, one could observe whether there are significant differences in performance in patient communication and emotional support.

## 5 Limitations

This study is limited to nursing interns in Inner Mongolia, and in the future, a large national sample study can be conducted to further confirm and gain a more comprehensive understanding of nursing interns’ empathy; the present study is a cross-sectional research design, which is limited in that it can only provide a certain temporal cross-section of the situation and cannot reveal in depth the temporal order and causal relationship between variables. In order to more comprehensively explore the dynamic development of empathy and other related variables and their interactions, future studies should consider adopting longitudinal research methods. In addition, the understanding of quantitative data can be further enriched and deepened by integrating qualitative research tools such as in-depth interviews or focus group discussions; it is important to note that this study used a convenient sampling method, which may introduce selectivity bias and thus have a potential impact on the accuracy and reliability of the findings. To avoid this bias, future studies should prioritize the use of random sampling method to select study participants. This will not only increase the representativeness and comprehensiveness of the sample, but also effectively reduce the bias caused by human selection factors, making the research results more scientific and convincing.

## 6 Conclusion

In this study, the empathy ability of nursing interns was classified into three categories, namely, humanistic care group, low empathy group, and high empathy group, through latent profile analysis; these three categories of empathy profile models have different influencing factors, therefore, from the perspective of nursing educators, according to the different characteristics of interns, differentiated interventions are adopted in the cultivation of interns, interns are positively guided to cultivate the concept of empathy, precision intervention not only plays a positive role in the pre-education of clinical internship, but also helps interns to correctly understand themselves, helps them to change their roles from interns to nurses, and lays a solid foundation for the establishment of a good patient–nurse relationship in the future, so that they can become creative and all-round development of nursing talents. In future studies, the researcher could further explore the following questions: (1) Is it possible to quantitatively analyze the specific impact of empathic competence on the quality of nursing care? (2) What specific educational methods or training activities are most effective in improving nursing interns’ empathy? (3) Does the empathic competence of nursing interns change with time, experience, and work environment? (4) Does high empathy make nursing interns more susceptible to burnout? How can we maintain high empathy while focusing on the mental health and job satisfaction of nursing interns?

The main contribution of this study is to bring a more refined approach to training in the field of nursing education, while emphasizing the centrality of empathic competence to the quality of care. By differentiating interventions and further exploring the association of empathic competence with quality of care and educational methods, this study points to new directions for future practice and research in nursing education.

## Data availability statement

The raw data supporting the conclusions of this article will be made available by the authors, without undue reservation.

## Ethics statement

The studies involving humans were approved by Ethics Committee of Inner Mongolia People’s Hospital. The studies were conducted in accordance with the local legislation and institutional requirements. The participants provided their written informed consent to participate in this study.

## Author contributions

JW: Conceptualization, Formal analysis, Methodology, Software, Validation, Visualization, Writing – original draft. XX: Conceptualization, Formal analysis, Methodology, Software, Validation, Visualization, Writing – original draft. JS: Data curation, Investigation, Resources, Writing – original draft. YM: Data curation, Resources, Writing – original draft. PT: Data curation, Investigation, Resources, Writing – original draft. WC: Conceptualization, Funding acquisition, Writing – original draft. XC: Investigation, Resources, Writing – original draft. YC: Investigation, Resources, Writing – original draft. MS: Conceptualization, Project administration, Supervision, Writing – review & editing. YH: Conceptualization, Project administration, Supervision, Writing – review & editing.
