# Supplementary material for: A study of latent profile analysis of empathic competence and factors influencing it in nursing interns: a multicenter cross-sectional study
Source: Front Public Health. 2024 Jun 26;12:1434089. doi: 10.3389/fpubh.2024.1434089 (PMC11234888; doi:10.3389/fpubh.2024.1434089)
Supplement: Supplementary file 1 [file Data_Sheet_1.pdf]

Supplementary Material

1 Sampling description

The Inner Mongolia region is divided into three regional distributions: eastern, central, and western, with a total of 12 cities in the Union. According to the workload, cost, and cooperation of the sample population, a total of 8 cities (among them, 3 cities are located in the eastern region, 2 cities are located in the western region, and 3 cities are located in the central region) and 11 Grade 3A general hospitals were sampled by non-equal-proportional stratified sampling method. A total of 532 interns were surveyed in 11 tertiary general hospitals using convenience sampling method, with 444 valid samples, and the questionnaire validity rate was 83.5%. According to the sample size estimation formula, the minimum sample size is 264, so this 444 cases sample can represent the nursing interns in the whole Inner Mongolia region's tertiary-level A-level general hospitals.
